# Evaluating Different Dimensions of Programme Effectiveness for Private Medicine Retailer Malaria Control Interventions in Kenya

**DOI:** 10.1371/journal.pone.0008937

**Published:** 2010-01-28

**Authors:** Timothy O. Abuya, Greg Fegan, Abdinasir A. Amin, Willis S. Akhwale, Abdisalan M. Noor, Robert W. Snow, Vicki Marsh

**Affiliations:** 1 Centre for Geographic Medicine Research, Kenya Medical Research Institute, Kilifi, Kenya; 2 Department of Epidemiology and Population Health, London School of Hygiene and Tropical Medicine, London, United Kingdom; 3 Malaria Control and Child Survival Department, Population Services International, Nairobi, Kenya; 4 Division of Vector-Borne Diseases, Ministry of Health, Nairobi, Kenya; 5 Kenya Medical Research Institute, University of Oxford, Nairobi, Kenya; 6 Centre for Clinical Vaccinology and Tropical Medicine, University of Oxford, Headington, Oxford, United Kingdom; Universidad Nacional Mayor de San Marcos, Peru

## Abstract

**Background:**

Private medicine retailers (PMRs) are key partners in the home management of fevers in many settings. Current evidence on effectiveness for PMR interventions at scale is limited. This study presents evaluation findings of two different programs implemented at moderate scale targeting PMRs for malaria control in the Kisii and Kwale districts of Kenya. Key components of this evaluation were measurement of program performance, including coverage, PMR knowledge, practices, and utilization based on spatial analysis.

**Methodology/Principal Findings:**

The study utilized mixed quantitative methods including retail audits and surrogate client surveys based on post-intervention cross-sectional surveys in intervention and control areas and mapping of intervention outlets. There was a large and significant impact on PMR knowledge and practices of the program in Kisii, with 60.5% of trained PMRs selling amodiaquine medicines in adequate doses compared to 2.8% of untrained ones (OR; 53.5: 95% CI 6.7, 428.3), a program coverage of 69.7% targeted outlets, and a potential utilization of about 30,000 children under five. The evaluation in Kwale also indicates a significant impact with 18.8% and 2.3% intervention and control PMRs selling amodiaquine with correct advice, respectively (OR; 9.4: 95% CI 1.1, 83.7), a program coverage of 25.3% targeted outlets, and a potential utilization of about 48,000 children under five. A provisional benchmark of 7.5 km was a reasonable threshold distance for households to access PMR services.

**Conclusions/Significance:**

This evaluation show that PMR interventions operationalized in the district level settings are likely to impact PMR knowledge and practices and lead to increased coverage of appropriate treatment to target populations. There is value of evaluating different dimensions of public health programs, including quality, spatial access, and implementation practice. This approach strengthens the potential contribution of pragmatic study designs to evaluating public health programs in the real world.

## Introduction

The popularity of private medicine retailers (PMRs) in the home management of fevers in many settings [1], [2] has led to the recognition of their importance for malaria control [3]. However the current evidence on programme effectiveness for PMR interventions at scale is limited, making it difficult to define public health policy for this sector. Moreover the legal status of PMRs varies, with each country having its own procedures and different categories of licenses for PMRs. Countries such as Tanzania have Part I and Part II pharmacies where Part I pharmacies are run by a registered pharmacist, and are allowed to sell both prescription-only and over-the-counter (OTC) medicines. Part II pharmacies also known as drug shops stock a variety of drugs [4]. In many settings including Kenya, most PMRs are licensed to sell over the counter (OTC) medicines such as analgesics and antipyretics. In practice however, many PMRs include other prescription only medicines such as antimalarials and antibiotics in their stock [5], [6].

Kenya has developed a number of malaria control initiatives targeting PMR practices and use of OTCs medicines in recent years. Here we present an evaluation of two of these programmes that encompassed different approaches to PMR interventions: One undertaken by the Ministry of Health (MoH) and the other by a Non-Governmental Organization (NGO) in two different parts of Kenya. The programmes were implemented between 2005 and 2007 as part of scaling up home management of malaria (HMM) strategy [7], supported in the Kenya National Malaria Strategy [8].

## Methods

### Broad Methodological Approach

In evaluating public health interventions, randomised controlled designs are regarded as gold standard. However, they may not be feasible, ethical or informative in a variety of situations, including complex interventions [9], [10], [11], [12]. Habicht and colleagues provide a useful pragmatic framework for identifying appropriate evaluation designs. They identify the ‘*what*’ is to be measured (provision, utilization, coverage and impact) and the ‘*how*’ or level of certainty for inference, from adequacy to plausibility and probability [9]. These pragmatic designs often incorporate the concept of plausibility instead of probability, aiming to construct a strong argument that a measured effect is unlikely to have been caused by inputs outside a given intervention. Key to the design is the selection of comparable control groups and rigorous assessment of the potential effect of any confounders.

The two PMR initiatives evaluated in this study represent examples of interventions with different elements, including training, demand creation and quality assurance, and requiring generation of an enabling environment. We have used Habicht's framework, including pragmatic elements dictated by practical limitations and addressing multiple dimensions of the interventions. Quantitative methods addressed impacts on PMR knowledge and practice and programme coverage and utilisation, while policy analysis of implementation experiences drew on qualitative methods. Here we focus on the quantitative elements while the policy analysis component is presented elsewhere (Abuya et al., submitted). Central to the quantitative analysis is the measurement of programme coverage, potential utilisation, and PMRs' knowledge and practices, allowing for a multidimensional perspective of potential public health impacts for these initiatives. The two PMR programmes were chosen as their different funding and management structures were of interest to the policy analysis component of the study. However, it is important to note that the design of the quantitative analysis component reported on in this paper does not allow for direct comparisons of these programmes, but for assessments of impact in each district.

### Overview of PMR Programmes Evaluated

The evaluation covered a programme supported by an NGO, Merlin (Medical Emergency Relief International), in Kisii Central district in Western Kenya and an MoH programme in Kwale district in Coast Province. The Kisii programme was characterised by a highland/epidemic type of malaria transmission [13] while the Kwale intervention was conducted in an area of endemic malaria transmission [13]. The Merlin PMR programme was implemented between 2005 and 2006 under a larger malaria control programme aiming to reduce morbidity and mortality associated with malaria through increasing capacity among formal and informal healthcare providers to diagnose and treat malaria according to the MoH guidelines. [14]. The Kwale programme was part of the first steps taken by the Division of Malaria control (DoMC) towards scaling up the HMM strategy in Kenya implemented in 2005. The programme aimed to train PMRs to stock MoH recommended anti-malarials, offer appropriate advice on the treatment of simple fevers in children with anti-malarials and educate community members on fever management [15]. Table 1 summarises key programme activities.

**Table 1:** Key Features of programme activities.

Characteristics	Kwale-MoH programme	Kisii-Merlin programme
Training	Two-day participatory training of PMRs on use of amodiaquine. Recruitment based on selling anti-malarial medicines, stability of outlet and positioning in remote settings. Training covered malaria treatment and control, signs and symptoms of malaria, signs requiring referral to trained health workers and communications skills	Three-day participatory training of PMRs on use of amoadiaquine. Recruitment based on selling antimalarial medicines, stability of outlet and positioning in remote settings, training covered malaria treatment an control, signs and symptoms of malaria, signs requiring referral to trained health workers, communication skills and record keeping
Demand creation	Public information activities through schools, churches and community distribution of T-shirts with messages on fever management	Not conducted
Accreditation	Paper posters	Wooden posters and award of certificates
Motivation	Financial token to actors participating in workshops. Per diem allowances of $ 3.7 given to PMRs and $ 5.2 for Public health officers and District health management teams members participating in the workshops	
Monitoring and evaluation	Follow ups not conducted	Visits to check records, administer quizzes and discuss ways of solving practical problems
Implementing agencies/funding	Ministry of health-Global Fund	NGO-Merlin in collaboration with ministry of health with funding from the government of Finland

### Study Design

The evaluations were conducted approximately six months post implementation in each district. Quantitative assessment of programme focussed on impact on PMR knowledge and behaviour, coverage and utilisation. The study designs differed between the programmes for practical reasons. A retrospective approach was used in Kisii since the programme was already in operation in Kiamokama division at the time of evaluation. A similar division (Suneka) was identified as a contemporaneous control where Merlin did not implement the programme. Two divisions in Kwale (Matuga and Kinango) had been randomly allocated to intervention area, while two others (Msambweni and Samburu) acted as controls [16]. In both sites, public health and administration leaders' knowledge of local malaria control activities, socioeconomic status, geographical features, malariometric indices and access to formal health facilities were used to identify divisions for the evaluation, either prospectively in the case of the MoH programme or retrospectively for the NGO programme.

### Study Methods

#### Global positioning systems (GPS) mapping and spatial analyses

To assess coverage and potential utilization, all outlets in the intervention areas were visited and mapped by field workers using GPS hand held receivers (Germin etrex and Trimble 12 band GPS units). Field workers then conducted a short interview on ownership of the outlet, type and brands of antimalarials available at the time of the survey and their involvement in the retailer programme. Thereafter, three coordinate readings of the outlets were recorded, each with the accuracy level of below 20 metres. Coordinates from GPS readings were entered in MicroSoft excel software and transferred to geographical information system (GIS) software Arcview GIS 3.2 (ESRI Inc., USA) to generate maps. The point data were overlaid with the polygon features of the intervention division to validate the geo-positioning of the outlets. To derive threshold distances for estimating utilisation, the proportion of clients using retail outlets for a fever in a particular locality was derived from a previous household survey [17]. Using the enumeration area (EAs) maps in the programme areas, distance in terms of walking time to visit each treatment source was derived using a surface model allowing for terrain [18].

The basic inputs for the model were journey time to the nearest retail outlet for fever treatment which was represented by a numeric code (1 for retail outlets and 2 for other options). The reference table was exported to S-Plus for widows 6.1 version (Insightful Corporation, Inc., Basingstoke, Hampshire, UK). The script calculated utilisation rate (UR) at each journey-time interval which was then smoothened by taking a moving average of 50 minutes for each point interval. The purpose was to assess the relationship between UR of retail sector users versus all other sources outside the home using a mathematical function used previously [18].

Once a threshold distance was derived, an under five population surface model was developed using the projected under five population for 2006 (based on the 1999 national census). In this analysis Thiessen Polygons (TP) were used to generate the catchment areas for each of the market centres (most outlets in this setting are either located in small or large commercial centres or in dotted around villages). TP assigned each point with a retail outlet and based on the threshold distance, polygon maps of outlet catchment areas were generated. A population surface for children under five covering a grid of 100 m by 100 m cell was used to estimate the number of children in each polygon. The under five population of all EAs within the centre's catchment area was summed as the potential users of the retail outlets.

#### Retail audit and surrogate client surveys

In the intervention areas of both districts, 80 study outlets were randomly sampled from a list of all trained outlets. The type of outlets available in these settings include general retail stores which sell a range of OTC medicines such as painkillers and some antimalarials alongside a range of household goods such as soap, cooking oil and groceries; general wholesale shops that supply goods to retail outlets; kiosks and market stalls; and itinerant hawkers who supply goods and medicines to outlets in the periphery. A sample size of 60 anti-malarial sales during the study period was calculated for the evaluation to demonstrate a 20% difference between control and intervention outlets with 80% power and 95% confidence intervals, from an estimated 5% of control outlets showing appropriate practice. An additional 20 sales were added to allow for data losses [19]. In the control group, a list of all outlets selling anti-malarial medicines was developed by local public health staff. Randomisation in control and intervention areas was conducted using STATA version 8 (Stata Corp, College Station, Texas, USA).

Six local field workers per division were recruited and trained for the surrogate client survey (SCS) and retail audit. The SCS was conducted first, during which field workers visited study outlets in both the control and intervention areas and requested an anti-malarial medicine for a child. Where asked for more information, they provided standardised responses to questions about the age of the child (three years), the symptoms of the illness (fever only) and any previous treatment given (none). Each visited outlets away from their own homes to avoid local recognition. Details of the transaction were entered in a simple form.

Following the SCS, a retail audit of all outlets visited during the SCS were conducted to collect information on general characteristics of the outlets and PMRs, drugs stocked; retailers' knowledge and reported practices through a vignette which sought to assess the selling practices on malaria medicines stocked; and their referral practices for a five year old child. Before each interview, the field workers gave a careful explanation of the purpose of the survey and sought verbal consent from the main seller. A pre-tested structured questionnaire was used to collect the information.

All the quantitative data from the surrogate client survey and retail audit were checked for errors and coded each day. Assessment of adequateness of advice of anti-malarial medicines was based on the national malaria guidelines. Data were double entered using FoxPro Version 6 software (Microsoft Corp, Redmond, USA). Verification, cleaning and analysis was conducted using STATA version 8 (Stata Corp, College Station, Texas, USA). Analysis within each district was done using chi–square tests of association to compare proportions for key outcome indicators. Where differences between the control and intervention areas were observed, a logistic regression model was used to estimate the magnitude of effect. The outcomes are presented as odds ratios derived from random effect model (using x*tlogit* command).

### Ethical Considerations

The study was approved by the Kenya National Scientific Steering and Ethical Research Committees (KEMRI SCC no 1056). Verbal informed consent was sought for the retail audit and mapping study. Given the nature of the method, informed consent could not be sought from PMRs for the surrogate client survey. Seeking consent for this study would have undermined the purpose of the study. Consent was thus obtained from community leaders and permission for an individual informed consent waiver was granted by the national ethics review committee.

## Results

### Programme Coverage

**Figure 1:**
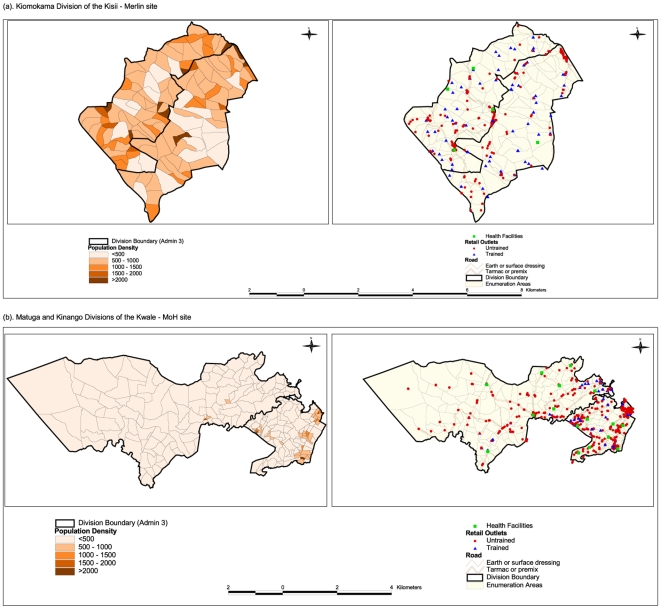
Maps of study sites showing population distribution, retail outlets, health facilities and transport networks. The first map shows projected population densities for 2006 in each enumeration area (EA). The second map combines retail outlets, health facilities and transport networks.

Figure 1 (a-b) shows the spatial distribution of retail outlets and health facilities in relation to population densities and transport networks. Population distribution in Kisii was more evenly distributed than that in Kwale (Figure 1a). The highest coverage of trained outlets was observed in Kiamokama division of Kisii, with 83/306 (27.1%) outlets trained compared to all divisions (Table 2). In Kwale, Figure 1.1b shows that most of the trained outlets were clustered around the coastal strip and major market centres. The programme covered 96/679 (14.1%) of all outlets with variations at the divisional level. However, Table 2 illustrates that overall many outlets in both districts did not stock anti-malarials (60.6% in Kisii and 43.9% in Kwale). The proportion of ‘true’ programme target outlets (that is, those stocking anti-malarials) reached was therefore 69.7% in Kisii and 25.3% in Kwale.

**Table 2:** Retail sector service indices across the programme sites.

Characteristics	Kisii site: Kiamokama division	Kwale site		
		Matuga division	Kinango division	Both divisions
***Coverage of programmes***				
Outlets in division	331	391	318	709
Open outlets/all outlets	306/331 (92.4%)	387/391 (98.9%)	291/318 (91.8%)	679/709 (95.8%)
Trained outlets/all open outlets	83/306 (27.1%)	42/387 (10.9%)	54/291 (18.5%)	96/679 (14.1%)
Outlets with anti malarial/open outlets*	119/302 (39.4%)	251/387 (64.9%)	128/291 (43.9%)	379/676 (56.1%)
Trained outlets/all outlets with antimalarials	83/119 (69.7%)	42/251 (16.7%)	54/128 (42.2%)	96/379 (25.3%)
***Health facilities and population distribution***				
Health facilities	9	4	4	8
Area	161 Km ^2^	341 Km^2^	1842 Km^2^	2183 Km^2^
Population projections by 2006†	121 844	86 284	86 405	172 689
Population density	757 persons/ Km ^2^	253 persons/Km^2^	47 persons/ Km ^2^	79 persons/ Km ^2^
Projected under five	18 226	12 781	15 897	28 678
Under five population potentially reached††	*29876*	8260	39575	47785
***Population service indices***				
PMR: population	1∶368	1∶220	1∶272	1∶243
PMR: under five population	1∶56	1∶33	1∶50	1∶40
Trained PMR: population	1∶1007	1∶2054	1∶1600	1∶1798
Trained PMR: under five population	1∶219	1∶304	1∶294	1∶299
Health facility: population	1∶13538	1∶ 21571	1∶∶10800	1∶21608
Health facility: trained PMR	1∶9	1∶9	1∶8	1∶12

*there were a number of outlets where information on anti-malarial medicines in stock could not be established, for example availability of anti-malarial in trained outlets could not be established in 2 outlets in Kisii, and 1 in Kwale respectively.

†population projections for 2006 are based on the 1999 census at an intercensal growth rate of 2.0% in Kisii and 2.6% in Kwale.

††This includes under fives living within 7.5 km of a trained outlet which may overlap with neighbouring areas.

### Potential Utilization of PMR Services

**Figure 2:**
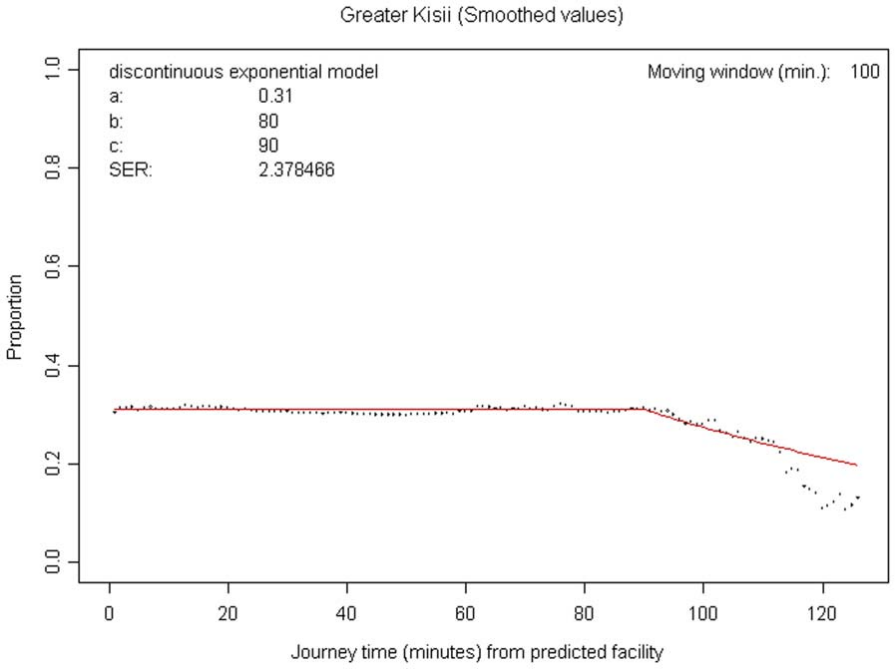
Graph showing utilization rates of retail sector services for treatment of fevers in the Kisii site. Summarises the output of the assessment of threshold distance to access retail sector services.

Results in this section were derived from modelling utilisation rates of retail outlets for fever treatment against all other treatment sources. Figure 2 illustrates the output of the analysis of threshold distance (considered as the minimum distance a care giver is likely to travel to access PMRs services). The y-axis shows the proportion of patients (under five) who used retail outlets at each interval while the x-axis shows predicted travel times in minutes to the outlets. The parameter C in Figure 2 represents the distance within which most caregivers would use the retail outlets. This parameter was 90 minutes, translating to a travel distance of 7.5 km (assuming a walking rate of 5 km/hour). The 7.5 km threshold distance was then used to estimate the underlying under five population likely to use trained retail outlets. Based on the threshold distance, the average travel distance to a centre with a programme outlet (trained outlet) was 1.30 km in Kisii and 1.86 km in Kwale. However, there were variations at divisional level in Kwale with Matuga division having an average travel distance of 1.05 km compared to 2.66 km in Kinango division. Taking both Kwale divisions together, the programme covered a potential target population of 47,785 children under five years. Potential utilisation in the remote rural division of Kinango (39,525 children) was higher than that for Matuga division (8,260 children) (Table 2). The Kisii programme (one division) had a potential utilisation of 29,876 children under five.

### Impact on Knowledge and Practices of PMRs

From the retail audit, 68.1% of intervention outlets in Kisii and 56.5% in the control areas stocked anti-malarial medicines. In Kwale these were 53.2% and 67.3% in the intervention and control areas respectively. These data differ from those shown for the GPS survey, since the sampling frame for the retail audit included target outlets for the programme and control outlets likely to stock anti-malarial medicines, from local knowledge. Most outlets surveyed were general retail shops run by one retailer. In terms of gender, education and age, PMRs were similar in the control and intervention areas in all sites. Most PMRs were relatively young (<35 years) and had between 8 and 12 years of schooling.

**Figure 3:**
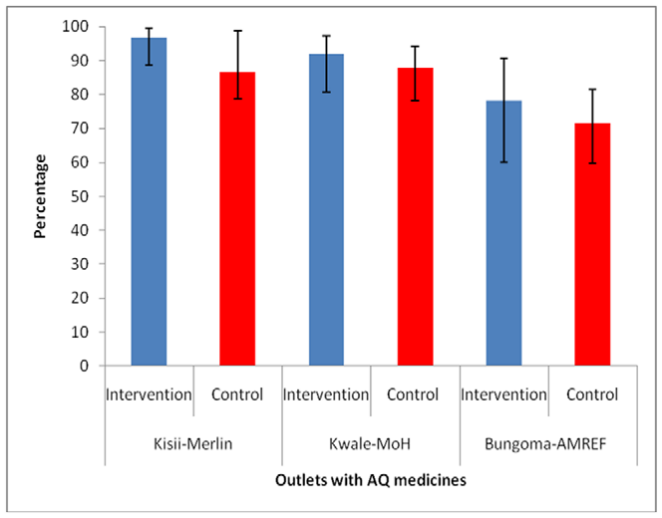
Proportion of outlets with anti-malarial medicines that stocked amodiaquine.

Figure 3 shows that a majority of all outlets with antimalarials stocked the MoH recommended drug, AQ, and this was not significantly different between control and intervention areas. In Kisii 96.7% of intervention outlets that stocked antimalarials stocked AQ medicines compared to 86.5% in the control area, p<0.023. In Kwale, these proportions were 92.0% in intervention and 87.8% in the control areas, p = 0.559. Table 3 illustrates levels of PMR knowledge on the use of OTC antimalarials. 79.3% of trained and 16.7% of control PMRs in Kisii knew the correct dosage for AQ (OR; 18.6: 95% CI 6.6, 52.2) while 48.8% of trained and no control PMRs were able to provide this information in Kwale. A common pattern for incorrect use in both districts was administration of AQ for one day. In both districts, there were no statistically significant differences between the control and intervention areas in the proportion of PMRs who had correct knowledge on the dosage of SP medicines.

**Table 3:** PMR's knowledge on dosing SP and AQ medicines for under fives.

Adequacy on dosages for anti-malarial medicines recommended	Kisii site				Kwale site			
	I n (%)	C n (%)	P	OR (95% CI)	I n (%)	C n (%)	P	OR (95% CI)
Dosing of AQ medicines available*								
Recommended AQ adequately	46/58 (79.3%)	7/42 (16.7%)	<0.001	18.6 (6.6, 52.2)	21/43 (48.8%)	0/58 (0%)	1.000	na
Recommended AQ in over dose	3/58 (5.2%)	17/42 (40.5%)	<0.001	0.08 (0.2, 0.3)	12/43 (27.9%)	18/58 (31.0%)	0.734	0.9 (0.3, 2.4)
Recommended AQ in under dose	8/58 (13.8%)	17/42 (40.5%)	0.002	0.2 (0.08, 0.6)	10/43 (23.3%)	34/58 (58.6%)	<0.001	0.2 (0.1, 0.5)
Recommended AQ for one day	8/58 (13.8%)	19/42 (45.2%)	<0.001	0.2 (0.07, 0.5)	3/43 (6.9%)	34/58 (60.3%)	<0.001	0.03 (0.004, 0.3)
***Dosing of SP medicines available***								
Recommended SP adequately	1/5 (20.0%)	8/20 (40.0%)	0.405	0.4 (0.04, 3.9)	11/23 (47.8%)	14/32 (43.7%)	0.765	1.2 (0.4, 3.4)
Recommended SP in high dose	4/5 (80.0%)	12/20 (60.0%)	0.621	2.7 (0.3, 28.4)	6/23 (26.1%)	10/32 (31.2%)	0.678	0.8 (0.2, 2.6)
Recommended SP in low dose	0/5	0/20	NA	NA	6/23 (26.1%)	8/32 (25.0%)	0.927	1.1 (0.3, 3.6)
Recommended SP for three days	4/5 (80.0%)	6/20 (30.0%)	0.121	9.3 (0.9, 101.2)	11/23 (47.8%)	3/32 (9.4%)	0.002	8.9 (2.1, 37.5)

Throughout this paper the following labels apply to all the tables. I  =  intervention area (Kiamokama in Kisii and Kinango and Matuga in Kwale); C =  control area (Suneka of Kisii and Msambweni and Samburu of Kwale)  =  P value for comparison of intervention and control in each site; OR  =  odds ratio of the comparison between control and intervention in each site.

†Use of NA -represents cases where that measure was not applicable or could not be derived.

Table 4 presents data on PMRs' anti-malarial selling practices. When asked by a surrogate client for an anti-malarial medicine for a young child, over half of PMRs sold anti-malarial medicines in all sites. Anti-malarial medicines were more often sold in the intervention compared to the control outlets in Kisii (OR; 4.4: 95% CI 1.1, 17.9) and Kwale (OR; 4.4: 95% CI 1.4, 13.8) but this was not statistically significant. As a proportion of all anti-malarial sales, 60.5% of trained PMRs sold AQ medicines accompanied with adequate advice on use compared to 2.8% of untrained PMRs in Kisii (OR; 53.5: 95% CI 6.7, 428.3). In Kwale, these percentages were 18.8% in the intervention and 2.3% in the control outlets (OR; 9.4: 95% CI 1.1, 83.1).

**Table 4:** PMR's practices while selling medicines for a three year old febrile child.

Selling practices	Kisii site				Kwale site			
	I (n) %	C (n) %	P	OR (95% CI)	I (n) %	C (n) %	P	OR (95% CI)
Sold anti-malarial with or without antipyretic*	40/43 (93.0%)	27/36 (75.0%)	0.032	4.4 (1.1, 17.9)	27/32 (84.4%)	23/42 (54.7%)	0.011	4.4 (1.4, 13.8)
Sold antipyretic only	3/43 (6.9%)	9/36 (25.0%)	0.026	0.2 (0.1, 0.9)	5/32 (15.6%)	19/42 (45.2%)	0.011	0.2 (0.1, 0.7)
***Type of anti-malarial sold and adequacy of dosage*** †								
Sold AQ	38/43 (88.4%)	20/36 (55.6%)	0.002	6.1 (1.9, 19.0)	19/32 (59.4%)	8/42 (19.1%)	<0.001	6.2 (2.2, 17.7)
Sold AQ with adequate advice	26/43 (60.5%)	1/36 (2.8%)	<0.001	53.5 (6.7, 428.3)	6/32 (18.8%)	1/42 (2.3%)	0.038	9.4 (1.1, 83.1)
Sold AQ with adequate advice on dose/all AQ sold	26/38 (68.4%)	1/20 (5.0%)	<0.001	41.2 (4.9, 344.4)	6/19 (31.6%)	1/8 (12.5%)	0.633	3.3 (0.3, 33.9)
Sold SP	2/43 (4.7%)	8/36 (22.2%)	0.037	0.2 (0.03, 0.9)	8/32 (25.0%)	15/42 (35.7%)	0.324	0.6 (0.2, 1.7)
Sold SP with adequate advice	0/36 (0%)	5/36 (13.8%)	0.017	NA	4/32 (12.5%)	8/42 (19.0%)	0.536	0.6 (0.2, 2.2)

*Note: the denominator was all medicines sold.

†the denominator is the outlets where either SP or AQ medicines were available at the time of the survey.

## Discussion

The main aim of this paper is to describe the potential contributions of two different PMR programmes to malaria control in Kenya, based on measurements of dimensions of performance (coverage, knowledge and practices of PMRs and potential utilisation). This evaluation indicates a large and significant impact on PMR knowledge and practices of the programme in Kisii, with 60.5% of trained PMRs selling AQ medicines in adequate doses compared to 2.8% of untrained ones (OR; 53.5: 95% CI 6.7, 428.3), a programme coverage of 69.7% targeted outlets and a potential utilisation of 29,876 children under five. In Kwale a significant impact was observed with 18.8% and 2.3% intervention and control PMRs, respectively, selling AQs medicines in adequate doses (OR; 9.4: 95% CI 1.1, 83.7), a programme coverage of 25.3% targeted outlets and a potential utilisation of 47,785 under fives. Of interest is the high potential utilisation in Kinango division of Kwale district (39,525 children), a remote rural area typical of sites where access to health care is of particular concern. We also suggest that a provisional benchmark of 7.5 km is a reasonable threshold distance for households to access PMR services, although further studies are important to examine this finding in different settings.

Use of different methods as part of this assessment provides a main strength for this evaluation. Further advantages include the use of contemporaneous controls for knowledge and practice surveys [4] and the close relationship between the intervention itself (training on over the counter anti-malarials) and the outcomes measured (knowledge and practice on the use of these anti-malarials). However, an important limitation is the timing of this evaluation, providing little evidence for sustainability of the changes measured. Data on sustainability remain scanty once the involvement of researchers or donors has ceased [4]. In addition, given the pragmatic nature of this evaluation, there are other potential limitations to interpretation that are important to consider.

### Pragmatic Approaches to Minimise Study Limitations

Randomisation and replication were either limited or not possible in these sites. In Kisii, this could not be achieved due to the retrospective nature of this evaluation of an on-going programme. In Kwale, while the evaluation could draw on a prospective cluster randomised design, the district level analysis was affected by the availability of only two interventions and two control areas. Use of a single survey was a further complication in interpreting the data. Without a historical control, it was not possible to establish whether differences (or similarities) between contemporaneous controls and interventions existed before the intervention, or result from the intervention.

In order to limit the effect of potential confounders, control areas were selected carefully taking into account local knowledge of factors considered important potential confounders, including access to formal health care, socio-economic factors, malaria burden and geographical location. Malaria, or at least fever, prevalence was a potential confounder, with higher levels being feasibly associated with greater use of anti-malarials, both at the outlet stocking and consumer levels, since these are related through demand. Although *Plasmodium falciparum* rates are very different in the two districts, at 67.5% in Kwale and 27.5% in Kisii, controls and interventions were chosen with similar rates in each district, as gauged by health officials from clinic and hospital records.

Data on the number of PMRs per outlet, gender, education and age of PMRs indicated comparability of the intervention and control areas in both districts. These factors, particularly educational status, were considered important potential sources of bias or confounding for an educational intervention. The number of PMRs per outlet was considered an important indicator of the size and economic viability of outlets [20], [21]. Similarities in all these features suggest that differences in the main outcomes measured were unlikely to have been influenced by different characteristics of the retail sector in these settings.

PMR practices were assessed through the SCS which, although frequently used and recommended for evaluating provider performance, is open to bias. The SCS is limited in terms of standardization of information, which potentially undermines the reliability of the results [22]. A further drawback of this method is inflexibility, including the inability to probe to uncover reasons behind any observed behaviours. For example, unexplained data loss was experienced during the SCS because of relatively high rates of PMRs refusing to sell over the counter medicines; (60.6% and 39.6% in Kisii; 41.6% versus 42.4% in Kwale from the intervention and control areas respectively). Reasons for these refusals were difficult for surrogate clients to explore without revealing their identity. Low numbers of observations in the SCS also limited the analysis, resulting from the low numbers of outlets trained, particularly in Kwale, the generally low levels of stocking anti-malarial and the tendency for PMRs to prefer not to sell anti-malarial medicines for young children; this finding being confirmed qualitatively. During the retail audit, response bias may have led to PMRs reporting practices that they assumed to be “desirable” rather than those they actually undertake, and thus to over reporting of correct practices. In addition, field workers were not blinded to the control or intervention group which may have influenced information recorded.

Efforts were made to minimise these sources of bias. Surrogate clients were trained using a skills based approach to support standardisation of information given to PMRs, data was collected on simple standardised collection forms and work reviewed every three days. To limit the chances of surrogate clients being recognised, field workers were recruited locally but visited outlets outside their normal locations. This meant working outside familiar areas and at times being forced to ask for directions. Although field workers were oriented with maps in advance, getting lost remained a logistical problem and a potential source of bias. All the above processes drew on experience with this method in other districts [19], [23].

There are also caveats to the interpretation of GPS mapping data and spatial analysis. Generally, using provider-population ratios ignores overlaps in potential border crossing between polygons by patients and instead assumes homogeneity in utility [24]. The approach does not incorporate measures of distance dimension [25] and spatial access was therefore measured using a Euclidean model. A limitation of this method is that catchment areas are delineated using TP techniques based on an assumption that patients choose the nearest retail outlet regardless of the type and services offered. However, in practice only a certain proportion will use retail sector services. Furthermore we did not have data from household data to examine actual utilization and factors influencing patient choice for different providers. This would have provided useful insights on how household level factors including patient choices may have influenced utilization.

### Implications for Policy

In common with previous research on interventions in this sector, and with the caveats described above, the study illustrates a potential for improving PMR behaviour through training and public information. A recent review showed that PMR interventions lead to greater knowledge, improved practices of PMRs and a higher rate of appropriate treatment of malaria and other childhood illnesses among communities [4]. For example, studies in Uganda indicate that the proportion of drugs stores giving appropriate drugs for uncomplicated malaria increased from 2% to 73% after negotiated sessions while those that recommended correct anti-malarial doses (CQ and SP) increased from 0% to 49% [26]. In Nigeria training and pre-packaging of antimalarials has been reported to lead to an increased proportion recommending correct anti-malarial doses from 9% to 53% [27]. In Kenya, 86% of PMRs gave appropriate advice on anti-malarial (CQ) after training compared to 0% on the untrained areas [23].

The study provides novel information on the potential programme coverage and utilisation, using modelling of GPS data. Use of spatial analysis in this study showed that despite low coverage of outlets and the highest physical distance to access a trained PMR, the Kwale site demonstrated a relatively high potential utilisation of target group compared to Kisii, and that this was primarily achieved in the more remote and sparsely populated division of Kinango. GPS mapping and spatial analysis have therefore provided insights into variations in the potential utilisation of programmes that are unable to be quantified from assessing simple proportions of outlets reached. However, further work is recommended on how spatial data can be used to assess the potential to reach different socio-economic groups.

Use of the TP approach also supports a more effective assessment of programme functioning than knowledge and practice surveys alone. For example, although the coverage of trained outlets with antimalarials may appear low in Kwale with only 25.3% of outlets with antimalarials being reached in Kwale and 69.7% in Kisii, the under five catchment population is likely to be high given the population distribution in these settings. Assuming community members use PMRs as a first source of treating simple fevers, if 60.5% of trained PMRs in Kisii sold AQ medicines in adequate doses, this would translate to about 18,074 children receiving adequate treatment if their caregivers sought care from trained PMRs. In comparison, in Kwale, 18.8% of trained PMRs sold AQ medicines adequately, translating into 8994 under fives who could receive adequate treatment. Such an assessment emphasises that ‘effective’ coverage needs a balance between coverage and quality of implementation.

Since 2007, the MoH first line recommended anti-malarial for use in the formal sector has been artemesinin combination therapy (ACTs). Pilot studies have been conducted in Kenya and elsewhere in sub Saharan Africa on their deployment in the private retail sector [28]. The findings on coverage in this study are of relevance to the debate on the feasibility and potential effectiveness of introducing ACTs into this sector. A key challenge for costly ACT medicines for the retail sector is affordability, both from a demand and supply perspective. Studies have shown previously that PMRs are often more likely to stock antipyretic than anti-malarial medicines for this reason [16], [23], [29], presenting a challenge to arguments that PMRs provide a sustainable channel to increase access to early effective treatment for malaria. The coverage data in this study suggests that a high proportion of even remote rural outlets can stock anti-malarial medicines (such as in Kinango), and that even the very low levels of programme implementation seen in these areas may support important utilisation for children under five years. This implies that the current commercial pricing of over the counter anti-malarials, such as AQ and SP, may in some situations act as a guide to the levels of subsidy needed for ACTs in this sector, and supports the current global efforts on subsidies [30].

As previously described, this study was not designed to compare outcomes quantitatively between the two programmes. The differences in knowledge gained, skills practised and outlet coverage do however look striking. Qualitative approaches, drawing on health policy analysis frameworks, support these as real differences and identify the likely contributing factors. These findings have been reported separately (Abuya et al, submitted) but, in summary, are linked to implementation gaps and challenges in managing the implementation process.

### Conclusions

Whilst it does not address sustainability, the study supports the findings of others in showing the potential for interventions based on participatory training and communication to improve the knowledge and skills of PMRs in selling anti-malarial drugs. A specific and relatively novel approach has been provided by using GIS as part of this evaluation. Spatial data have highlighted specific challenges around programme coverage as well as indicating the potential usefulness of even relatively low coverage levels in remote rural areas, depending on patterns of population distribution. While needing further research to support this finding, the technique proposes a specific distance of 7.5 km that could be used in measuring access to programme outlets. The spatial analysis of antimalarial coverage in both control and intervention outlets in these two districts also provides some evidence for price setting for subsidised ACTs in future, and supports current Affordable Medicines for malaria (AMFm) strategies in other malaria endemic countries. Overall, the study illustrates the value of using different dimensions of programme functioning to assess performance, including quality, spatial access and implementation practice. This multi-dimensional strengthens the contribution of pragmatic study designs to evaluating public health programmes in the real world.
